# The Ohio Medical and Surgical Journal

**Published:** 1848-10

**Authors:** 


### THE OHIO MEDICAL AND SURGICAL JOURNAL.

This is the title of a new medical periodical, to be published every other month at Columbus, in Ohio. Each number is to contain ninety-six pages, octavo.

The first number, which is now before us, bears date, September, 1848. It is neatly printed, on good paper, and contains a large variety of useful matter, both orignal and selected. The work is to be under the editorial management of John Butterfield, M. D., Professor of the Practice of Medicine in the Starling Medical College, recently established at that place, aided by his colleagues.

The editor professes to have had no experience in his new employment, but the spirit of enterprise is in him; and, judging from the specimen before us, and the reputation he has already won in other professional undertakings, we entertain no doubt of the success of his efforts, and that our new contemporary will take good rank among the medical periodicals of the country. With this expression of our feelings, we add it with pleasure to our list of exchanges.

